# Infection of the Jackal (*Canis aureus*) by *Haplorchis taichui* (Trematoda: Heterophyidae) in Southwestern Iran: A Clue for Potential Human Infection

**Published:** 2019

**Authors:** Salma TEIMOORI, Gholamreza MOWLAVI, Yuji ARIMATSU, Banchob SRIPA, Iraj MOBEDI, Meysam SHARIFDINI, Jafar MASSOUD, Saied Reza NADDAF

**Affiliations:** 1. Center of Excellence for Therapeutic Proteins and Antibody Engineering, Department of Parasitology, Faculty of Medicine, Siriraj Hospital, Bangkok 10700, Thailand; 2. WHO Collaborating Center for Research and Control of Opisthorchiasis, Tropical Disease Research Laboratory, Department of Experimental Pathology, Faculty of Medicine, Khon Kaen University, Khon Kaen 40002, Thailand; 3. Department of Parasitology and Mycology, Tehran University of Medical Sciences, Tehran, Iran; 4. Department of Medical Microbiology, School of Medicine, Guilan University of Medical Sciences, Rasht, Iran; 5. Department of Parasitology, Research Center for Emerging and Reemerging Diseases, Pasteur Institute of Iran, Tehran, Iran

**Keywords:** Heterophyidae, *Haplorchis taichui*, *Canis aureus*, ITS2, Iran

## Abstract

**Background:**

We detected eight trematodes in the small intestine of a road-killed jackal (*Canis aureus*) from Hamidiyeh District near the city of Ahvaz, Khuzestan Province in 2010.

**Methods:**

Three worms were stained with carmine acid, mounted in Canada balsam on glass slides and examined under a light microscope at 1000X magnification. PCR and sequencing of a partial ITS2 sequence were used to approve the diagnosis.

**Results:**

The flukes measured ≈1 mm in length with an elongated ovoid shape resembling the members of heterophyid, and only one testis, characteristics of the genus *Haplorchis.* Sequencing of a 481-bp fragment of the ITS2 locus from the worms revealed 97%–98% identity with the similar sequences of the *H. taichui* flukes previously identified in the fish, cat, and humans from Thailand, China, and Vietnam.

**Conclusion:**

Further studies with the application of reliable molecular tools to diagnose trematode infections in wildlife and humans can bring more insight into the epidemiology of fish-borne flukes including *H. taichui* in this area.

## Introduction

Foodborne disease (FBD) is a pervasive public problem worldwide caused by the consumption of contaminated food and drink. Pathogens including bacteria, viruses, and parasites as well as poisonous chemicals and other harmful substances entering the body through contaminated food or water can cause foodborne illnesses (1). Some water- or food-borne diseases, predominantly bacterial infections, are quickly diagnosed and reported more frequently as they appear in clusters with overt clinical manifestations, while foodborne parasitic diseases (FBPDs) have received less attention and mostly remain underdiagnosed (2). The FBPDs, mainly those caused by helminths seem to be on the increase due to the globalization of the food supply and improvement of detection methods (3).

Today, in Iran, the primary cause of FBPDs is the consumption of raw herbs and vegetables commonly served at Persian tables. Eggs of various parasitic helminths including *Fasciola* spp., *Dicrocoelium* spp., *Ascaris lumbricoides*, *Toxocara* spp., *Trichostrongylus* spp., and taeniids were frequently recovered from the herbs and vegetables offered in the suburban and rural daily markets (4, 5). Most of the helminths infection mentioned above show a downward trend (6), while human fascioliasis remains a public health concern in many parts of Iran especially along the Caspian Sea littoral (7, 8).

The meat-borne helminth infections such as *Taenia solium*-associated taeniasis, and trichinellosis (despite the occurrence of *Trichinella* spp. in the wildlife) are not considered a threat as the Islamic codes prohibit consumption of swine meat in the country. *T. saginata* infections with as low prevalences as ≤ 1% in the cattle (9) are scarce due to the strict inspection of carcasses at slaughterhouses (10). In Iran, the freshwater fish-borne helminthic infections are presumably limited to Khuzestan Province, southwestern of the country, where tributary slow running streams of effluent rivers of Karoun and Karkhe and marshlands provide suitable habitats for varieties of freshwater fishes and snails (11, 12).

In the present study, we report infection of a jackal (*Canis aureus*) with a fish-borne trematode, *Haplorchis taichui* (Trematoda: Heterophyidae) in southwestern Iran. We also discuss the presence of this fluke in the wildlife of this area and the possible occurrence of *H. taichui* human infections. Moreover, we speculate the habits and traditional practices that may probably contribute to fish-borne trematode infections in this region of the country.

## Materials and Methods

### Sample collection

**Fig. 1:**
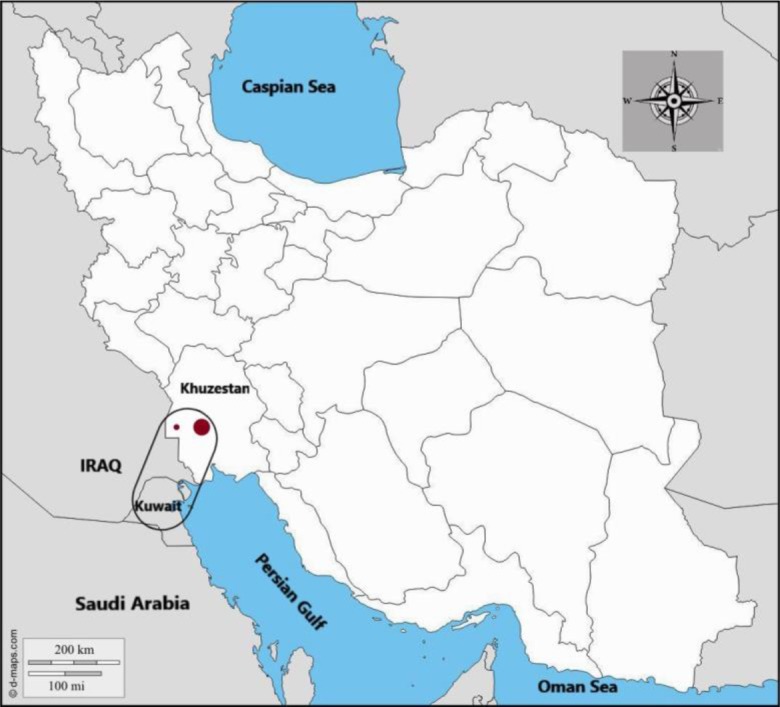
*Haplorchis taichui* in wildlife of southwest Asia (southwestern Iran, Kuwait, and southeastern Iraq). The big red dot indicates the city of Ahvaz and the small red dot the Hamidiyeh County where the *H. taichui-*infected Jackal was found

In 2010, we encountered a road-killed Jackal (*Canis aureus*) in Hamidiyeh District (31.478127, 48.433485), Khuzestan Province, southwestern Iran (Fig. 1). After dissection, eight minuscule worms were recovered from the small intestine of the animal. The worms were individually preserved in ethanol (80%) for further morphological and molecular investigations. No other helminth species was found in the intestinal lumen of the jackal.

### Morphological identification

Three worms were stained with carmine acid, mounted in Canada balsam on glass slides as described elsewhere (13) and examined under a light microscope with a magnification of 1000X.

### PCR and Sequencing

Genomic DNA was extracted from two individual worms using a commercial QIAamp DNA Mini kit (QIAGEN, Germany) as recommended by the manufacturer. A partial fragment of ITS2 sequence was amplified using the primers that yield amplicons of variable sizes for the species *Opisthorchis viverrini*, *Clonorchis sinensis,* and *H. taichui* as well as other species of opisthorchiid and heterophyid flukes (14). The PCR reactions contained 2 μl of 10X buffer, 200 μM of dNTPs, 100 pmol of each primer, 2 μl of 1:10 diluted DNA, 0.5 U of Pfu DNA polymerase (Promega, USA), and double distilled water to a final volume of 20 μl. The amplification program consisted of a 1-minute initial stage at 95 °C, followed by 35 cycles of denaturing at 95 °C for 30 sec, annealing at 60 °C for 30 sec, and extension at 72 °C for 2 min, and a final extension at 72 °C for 5 min. The amplicons were resolved on a 1% agarose, stained with ethidium bromide in TAE buffer and visualized under UV.

The amplicons were purified using Nucleo-Spin® Gel and PCR clean-up (MARCHEREY_NAGEL GmbH & Co. KG, Germany), cloned into a pJET1.2 cloning vector and transformed into TOP 10 *E. coli* competent cells.

The transformed cells were plated on a selective agar medium culture containing 100 μl/ml of ampicillin. Plasmids from single colonies of bacteria, grown overnight in LB medium, were purified using GeneJET™ Plasmid Miniprep Kit (Thermo Scientific, USA), and sequencing of the cloned gene was performed by 1^st^ Base Asia, Malaysia (http://www.base-asia.com/) using the Sanger method. The generated sequences were BLASted against similar sequences available in the GenBank database, and one sequence as a representative was submitted to the GenBank database under the accession number MF043945.

## Results

### Morphological description

**Fig. 2:**
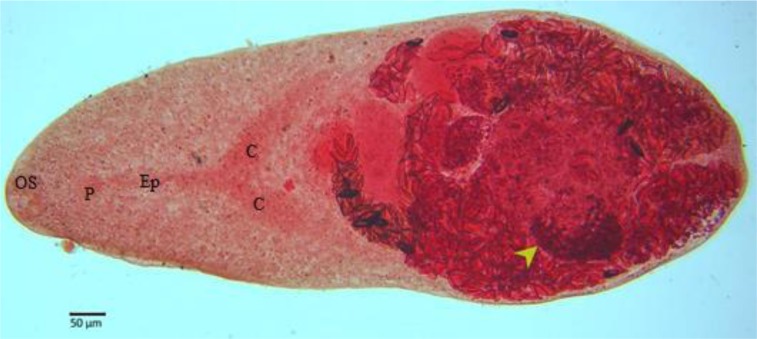
The fluke recovered from a Jackal (*Canis aureus*) in Khuzestan Province. The “OS” shows oral sucker, “P” pharynx, “Ep” esophagus, “C” cecal branches, and the arrow (➝) the single testis, a characteristic feature of the genus *Haplorchis*

The helminths measured about 1 mm in length with an elongated ovoid shape resembling heterophyid flukes. The oral sucker (OS), pharynx (P), long esophagus (Ep), and cecal branches (C) at the anterior part of the trematode were prominent with the broadest part (370 μm) at the posterior of the body. The single lobed testis, characteristics of the genus *Haplorchis* (≈120 μm in diameter) was near to the posterior end of the worm. The ovaries were hardly visible, but many light brown ≈25×14 μm eggs were visible in the uterus. Valid identification of the species was not possible as the distinctive features, i.e., the genital sucker, and the arrangement of the spines in the ventro-genital complex were not recognizable (Fig. 2).

### PCR and Sequencing

Amplification of the ITS2 locus yielded the expected 520 bp amplicon. The 481bp sequence generated in this study matched 97–98% (100% coverage) with the similar sequences of the adult *H. taichui* flukes (accession numbers HM004155, HM004156, HM004157, KP165440, KC999100, and KX815126) identified in fish, cat, and humans from Thailand, China, and Vietnam.

## Discussion

Heterophyid flukes are small intestinal trematodes measuring ≈ 0.5–2 mm in length. They can survive and reproduce in a wide range of animal hosts (15). Humans acquire infection via eating raw or undercooked as well as pickled and smoked fishes containing infective metacercariae. High human infection rates in the east and Southeast Asia is linked to the popularity of raw or undercooked freshwater fishes, served in local cuisines such as sushi (16, 17). Previously, there were limited reports of these fish-borne fluke infections, but with the improvement of detection methods, infection rates as high as 36% were reported in humans from southern Philippines (18). In Iran, two studies reported heterophyid eggs in the fecal samples of people residing in the rural, swampy areas of Khuzestan Province, and the described the species, *Metagonimus yokogawai*, *Heterophyes heterophyes,* and *H. katsuradai* in the canids (6, 19). Later, in the same area, the metacercariae of *H. taichui* and other heterophyids, *Haplorchis pumilio*, *Haplorchis taichui*, *Stellantchasmus falcatus* and *Centrocestus formosanus* were detected in various fish species (11, 12), and different birds showed to harbor *H. taichui* flukes along with other trematodes (20). In the neighboring country of Kuwait, *H. taichui* causes infection in stray cats (21), and in the south of Iraq, this parasite occurs in the wildlife (18). Iraq and Kuwait share the Mesopotamian Marshlands (Horolazim wetland) with Iran. This wetland, once used to be the largest ecosystem of western Eurasia, is home to various species of animals and fish, and support the enzootic cycle of various fish-borne flukes.

Unlike Southeast Asia, raw or undercooked fish is not a favorite meal in Iran. Human infections in Khuzestan Province (6, 19) is assumed to occur during gutting and scaling of the fish that result in metacercariae contamination of hands, and the ingestion of this infective stage via the hand-to-mouth contact. A traditional misbelief, which prescribes swallowing live small fishes or the fingerlings for the treatment of jaundice (22) may also contribute to human infections. Despite the occurrence in the wildlife, no definitive report of human infection with *Haplorchis* species is available from Khuzestan Province. The morphology and sizes of the heterophyid eggs previously detected in the human fecal samples from this area were not discriminative enough to identify the species with certainty (23). Therefore, *H. taichui* eggs, despite their possible occurrence in the human feces might have remained undiagnosed. Accounts of similar fish-borne helminthic infections are available from Khuzestan Province. The eggs, larvae, and adults of *Capillaria philippinensis* were found in feces of a 30-year old fisherman residing in a village near to Ahvaz (24).

The worms recovered in the present study showed 97–98% identity with the *H. taichui* flukes previously identified in Thailand, China, and Vietnam. The reason for the delayed molecular analysis of the specimens was the lack of access to the appropriate instruments. An exhaustive study with the application of molecular tools to discriminate between the fluke species (25, 26) can bring more insight into the epidemiology of heterophyid infections including *H. taichui* in southwestern Iran.

## Conclusion

The wildlife of Khuzestan Province in southwestern Iran supports the life cycle of various species of heterophyid flukes including *H. taichui.* In this province, people, particularly those in rural areas have immediate access to freshwater fishes, which might put them are at the risk of fish-borne infections. During the last two decades, considerable changes in the ecosystem of the area by human activities, e.g., dam construction and drying of wetlands have affected the habitats of freshwater snails and fishes, and inevitably the incidence and distribution of the heterophyid flukes in the area. These flukes, though primarily described as lumen dwelling parasites, might spread beyond intestine and cause severe health problems. The minute eggs of the intestinal flukes, e.g., *Heterophyes heterophyes* are capable of passing through the intestinal wall and reaching the organs, like the brain, spinal cord, and heart via bloodstream (27). Hence, in endemic areas like Khuzestan Province, fish-borne trematodes merit more attention and should be considered beyond just enteric diarrheal diseases.
